# Roles of endoplasmic reticulum stress in the pathophysiology of polycystic ovary syndrome

**DOI:** 10.3389/fendo.2023.1124405

**Published:** 2023-02-15

**Authors:** Hiroshi Koike, Miyuki Harada, Akari Kusamoto, Zixin Xu, Tsurugi Tanaka, Nanoka Sakaguchi, Chisato Kunitomi, Jerilee M. K. Azhary, Nozomi Takahashi, Yoko Urata, Yutaka Osuga

**Affiliations:** ^1^ Department of Obstetrics and Gynecology, Faculty of Medicine, The University of Tokyo, Tokyo, Japan; ^2^ Department of Obstetrics and Gynaecology, Faculty of Medicine, University of Malaya, Kuala Lumpur, Malaysia

**Keywords:** endoplasmic reticulum stress (ER stress), pathophysiology, polycystic ovary syndrome (PCOS), unfolded protein response (UPR), follicular microenvironment, ovary

## Abstract

Polycystic ovary syndrome (PCOS) is the most common endocrine disorder among reproductive-age women, affecting up to 15% of women in this group, and the most common cause of anovulatory infertility. Although its etiology remains unclear, recent research has revealed the critical role of endoplasmic reticulum (ER) stress in the pathophysiology of PCOS. ER stress is defined as a condition in which unfolded or misfolded proteins accumulate in the ER because of an imbalance in the demand for protein folding and the protein-folding capacity of the ER. ER stress results in the activation of several signal transduction cascades, collectively termed the unfolded protein response (UPR), which regulates various cellular activities. In principle, the UPR restores homeostasis and keeps the cell alive. However, if the ER stress cannot be resolved, it induces programmed cell death. ER stress has recently been recognized to play diverse roles in both physiological and pathological conditions of the ovary. In this review, we summarize current knowledge of the roles of ER stress in the pathogenesis of PCOS. ER stress pathways are activated in the ovaries of both a mouse model of PCOS and in humans, and local hyperandrogenism in the follicular microenvironment associated with PCOS is responsible for activating these. The activation of ER stress contributes to the pathophysiology of PCOS through multiple effects in granulosa cells. Finally, we discuss the potential for ER stress to serve as a novel therapeutic target for PCOS.

## 1 Introduction

Polycystic ovary syndrome (PCOS) is the most common endocrine/metabolic disorder in women of reproductive age and presents with various symptoms, including oligomenorrhea, infertility, cutaneous manifestations, and metabolic disorders (1). Although its etiology remains unclear, recent research has revealed that intraovarian local factors play crucial roles in the pathophysiology of PCOS (2). The ovary is a dynamic organ: it is a site for follicular growth, oocyte maturation, ovulation, and corpus luteum formation in women of reproductive age. The ovarian follicular microenvironment, which includes gonadotropins and intraovarian local factors, is a key regulator of this dynamic process. Endoplasmic reticulum (ER) stress has recently been recognized to be a local factor in the follicular microenvironment (3). ER stress is caused by the accumulation of unfolded or misfolded proteins in the ER, and induces the activation of several signaling cascades that mediate its resolution and restore cellular homeostasis but also induce programmed cell death if this resolution is not possible. ER stress plays crucial roles in various pathological conditions and recent studies have shown that ER stress is involved in the pathogenesis of PCOS (1, 3, 4). To improve the healthcare of women with PCOS, it is important to better understand the pathophysiology of PCOS. In this review, we summarize current knowledge of the roles of ER stress in the pathogenesis of PCOS.

## 2 Polycystic ovary syndrome

PCOS is the most common endocrine or metabolic disorder in reproductive-age women and presents with heterogeneous and complex symptoms (1, 5, 6). For its diagnosis, the 2003 Rotterdam criteria are widely used. These require at least two out of the following three characteristics: clinical and/or biochemical hyperandrogenism (HA), ovulatory dysfunction, and polycystic ovarian morphology (7). The metabolic dysfunction related to insulin resistance (IR) is also a feature of PCOS, although IR is not included in the listed criteria (8).

HA and IR are the most important contributors to the pathophysiology of PCOS (1). HA disturbs the function of hypothalamic-pituitary-ovarian (HPO) axis: it causes abnormal gonadotropin-releasing hormone (GnRH) secretory pulses, resulting in high levels of luteinizing hormone, which further increases androgen secretion by ovarian theca cells (TCs) (9). Abnormal secretion of both gonadotropins and androgens disturbs ovarian function, including follicular development, steroid hormone secretion, oocyte maturation, and ovulation. In addition, high levels of anti-Müllerian hormone (AMH), which is secreted by the pre-/small antral follicles that accumulate in the ovaries of women with PCOS, further worsens ovarian dysfunction by disturbing follicular development and GnRH pulsation (10, 11). IR and the compensatory hyperinsulinemia that are associated with visceral adiposity and adipocyte dysfunction further aggravate the HA and ovarian dysfunction (10).

Although the familial nature of PCOS has been recognized for decades, it is estimated that the genes responsible for the pathogenesis of PCOS account only for 10% of its heritability (12). Instead, it is now considered that PCOS is a multifactorial disorder that is influenced by environmental factors, such as the prenatal intrauterine environment of mothers, the follicular microenvironment of the ovary, and lifestyle following birth (1, 5, 13). Prenatal exposure to high concentrations of androgens, AMH, or insulin in the uterus of mothers with PCOS may contribute to the pathogenesis of PCOS. ER stress, the proinflammatory status, and oxidative stress in the follicular microenvironment are likely to be responsible for the ovarian features of PCOS, as discussed in the following sections in detail. In addition, an unfavorable lifestyle predisposes toward the development of the metabolic features of PCOS.

## 3 Endoplasmic reticulum stress

**Figure 1:**
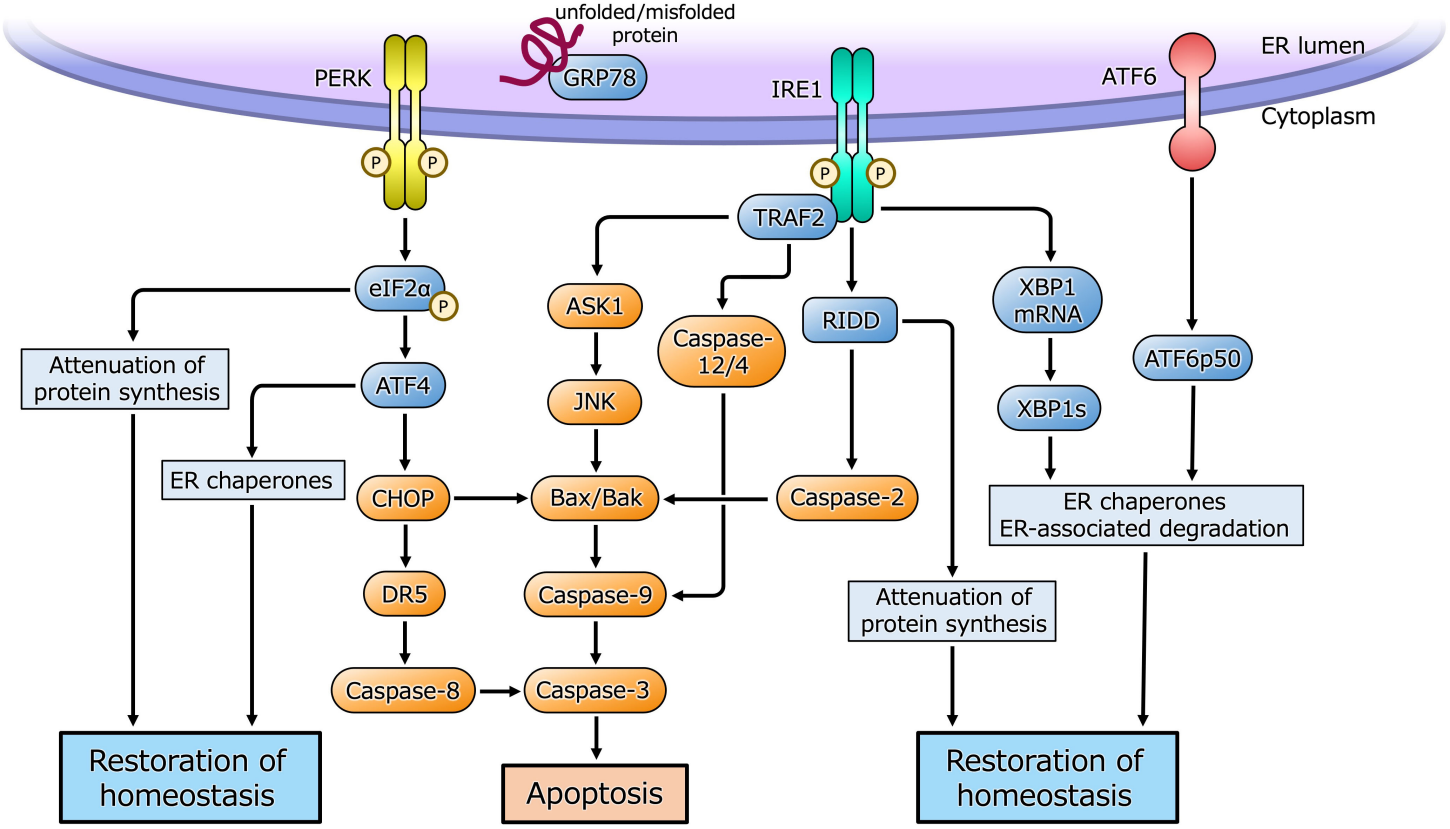
ER stress and the UPR pathway. Under ER stress, three sensor proteins located in the ER membrane are activated. Double-stranded RNA-activated protein kinase (PKR)-like ER kinase (PERK) and inositol requiring enzyme 1 (IRE1) are maintained in an inactive state by interaction with the ER chaperone glucose-regulated protein 78 (GRP78), and are activated by dimerization and self-phosphorylation when GRP78 binds to unfolded or misfolded proteins in the ER and is released from PERK and IRE1. PERK phosphorylates eukaryotic initiation factor 2α (eIF2α) that causes translational arrest of most proteins to reduce overload of protein folding in the ER while facilitates translation of activating transcription factor 4 (ATF4). ATF4 induces the transcription of its target genes encoding factors involved in the ER chaperones, amino acid biosynthesis, oxidative stress response and apoptosis. CHOP induced by ATF4 activates apoptotic cascade through the induction of DR5 or Bcl-2-associated X protein (Bax)/Bcl-2 homologous antagonist/killer (Bak). IRE1 is activated by dimerization and self-phosphorylation causing X-box-binding protein 1 (XBP1) mRNA splicing *via* its endoribonuclease activity. Spliced XBP1 (XBP1s) protein induces transcription of UPR target genes involved in the ER chaperones, ER-associated degradation and cell homeostasis. IRE1 also cleaves ER-associated mRNAs and non-coding functional RNAs, resulting in their degradation through regulated IRE1-dependent decay (RIDD), which reduces protein folding load in the ER or activates apoptotic cascade. IRE1 also binds to adaptor protein, tumor necrosis factor receptor-associated factor 2 (TRAF2), and activates apoptotic cascade through c-Jun N-terminal kinase (JNK) or caspase-12/4 signaling. Activating transcription factor 6 (ATF6) is activated by sequential cleavage in Golgi apparatus, releasing its active fragment, ATF6p50, which induces transcription of UPR target genes involved in the ER chaperones, ER-associated degradation and cell homeostasis.

The ER is a eukaryotic organelle that is principally involved in the synthesis, folding, maturation, and transportation of proteins, as well as in calcium homeostasis, lipid metabolism, and steroid synthesis. Cellular homeostasis is maintained by the competence of the ER to participate in these processes. However, under certain situations, such as cell proliferation and differentiation, hypoxia, or metabolic abnormalities, an imbalance between the functional capacity of the ER and the load causes cells to enter the state referred to as ER stress (14–17). ER stress causes the activation of a cellular adaptive mechanism, termed the unfolded protein response (UPR), to restore cellular homeostasis (**Figure 1**). However, severe or long-lasting stress causes a switch from the adaptive UPR to the maladaptive UPR, ultimately leading toward apoptosis. Because of its essential roles in diverse cellular activities, ER stress and the UPR have been implicated in various pathological conditions, such as diabetes, neurodegenerative diseases, inflammatory diseases, and various types of malignancy (18–23). Furthermore, recent studies have shown that ER stress is closely associated with benign gynecological diseases, such as PCOS (1, 3, 4).

## 4 Pathophysiological role of ER stress in PCOS

Ovarian follicles consist of an oocyte, granulosa cells (GCs), and TCs. Follicular development is a well-coordinated process that includes oocyte maturation and the proliferation and differentiation of somatic cells, which are regulated by gonadotropins and intraovarian local factors that affect the follicular microenvironment (24, 25). Recently, it has been revealed that ER stress in the follicular microenvironment, followed by activation of the UPR, plays an important role in ovarian physiology and pathology (3, 22, 26). HA, IR, inflammation, and oxidative stress are all associated with PCOS and closely connected with ER stress (1, 23). We demonstrated for the first time that ER stress and the UPR are activated in GCs from both patients with PCOS and mice with androgen-induced PCOS (4). We also demonstrated that a high androgen concentration induces ER stress in cultured human GCs (27). It is conceivable that ER stress and several local factors form a vicious circle within the follicular microenvironment of patients with PCOS and contribute to the pathophysiology (1).

### 4.1 Apoptosis of GC

Bidirectional communication between GCs and oocytes, as well as between somatic cells, regulates follicular development and oocyte maturation (28–32). These interactions are mediated *via* cell-to-cell gap junctions and in a paracrine or juxtacrine manner (33–36). Disruption of intrafollicular crosstalk disturbs normal follicular development and results in follicular atresia. On the basis of these findings, it is thought that inappropriate apoptosis of GCs disturbs follicular development. Indeed, apoptotic GCs are more numerous in ovaries from patients and animal models with PCOS (37, 38). Androgens induce the apoptosis of cultured GCs by activating intrinsic apoptotic signaling or reducing the synthesis of follicular growth factors (39–41).

ER stress induces the expression of the proapoptotic factors C/EBP homologous protein (CHOP) and death receptor 5 (DR5), which is high in the GCs of patients with PCOS (27). Treatment of cultured human GCs with testosterone activates several UPR pathways and DR5 and increases the apoptosis of these cells. These effects are attenuated by treatment with an ER stress inhibitor tauroursodeoxycholic acid (TUDCA). Treatment with TUDCA also attenuates the intrinsic apoptotic pathway, which is activated in the GCs of patients with PCOS (42). Furthermore, the administration of TUDCA to mice with PCOS reduces the apoptosis of GCs in antral follicles, and this is associated with a concomitant decrease in the expression of CHOP and DR5, which implies that ER stress activated by HA in PCOS promotes the apoptosis of GCs *via* CHOP followed by caspase cascade and follicular growth arrest (27). Similarly, treatment with curcumin, a polyphenol extracted from turmeric rhizomes, or traditional Chinese medicines abrogates androgen-induced ER stress and the apoptosis of GCs, both *in vivo* and *in vitro* (43–46). In addition, the high levels of oxidative stress that is founded in patients with PCOS cause the activation of the UPR in both the ER and mitochondria, resulting in the apoptosis of GCs (47).

### 4.2 Insulin resistance

IR and the resulting compensatory hyperinsulinemia are key components of PCOS pathophysiology, along with HA. IR is associated with significant alterations in ovarian function (48–51). Furthermore, hyperinsulinemia facilitates androgen secretion from the ovary and the adrenal gland and inhibits the hepatic synthesis of sex hormone-binding globulin, thereby increasing both total and free circulating androgen concentrations (52–54). In addition, HA induces visceral adiposity and adipocyte dysfunction, which cause IR. Therefore, IR and HA form a vicious circle. It has been proposed that ER stress underlies the development of IR by inhibiting insulin signaling in adipose tissue, skeletal muscle, and the liver (55–58). Pancreatic β-cells contain a large amount of ER, reflecting their insulin secretory function, and overwhelming demand for insulin secretion inevitably causes ER stress and β-cell dysfunction. Because of these findings, we will focus on the relationships among ER stress, IR, and HA in the pathophysiology of PCOS.

Both ER stress and the levels of proapoptotic factors are high in the pancreatic islets of mice with PCOS (59). Testosterone induces both of these abnormalities and the apoptosis of cultured islet cells, while the ER stress inhibitors TUDCA and 4-phenylbutylic acid (4-PBA), and the androgen receptor antagonist flutamide, ameliorate testosterone-induced ER stress and apoptosis in these cells (60). Moreover, the administration of flutamide to mice with PCOS ameliorates their hyperinsulinemia and the ER stress in their islet cells. It has been suggested that HA induces β-cell dysfunction *via* the activation of ER stress in PCOS. Furthermore, several studies have shown that ER stress distant from the pancreas may also be involved in the pathogenesis of PCOS. The phosphatidylinositol-3 kinase (PI3K)/Akt pathway, which is a major insulin signaling pathway and decreased in PCOS patients, and the UPR in GCs are involved in the dysfunction of these cells (44). It is also thought that kisspeptin, a peptide that regulates the HPO axis, is involved in IR and ER stress in the hypothalamus of individuals with PCOS (61). The administration of curcumin; traditional Chinese medicines; or adrenomedullin, an endogenous vasodilator peptide, ameliorates IR, which is accompanied by a concomitant decrease in ER stress in the ovaries of animals with PCOS (44, 45, 62, 63). Accordingly, it is conceivable that ER stress activated by HA in PCOS, both in islet and nonpancreatic cells, contributes to the induction of IR, and IR in turn activates ER stress in these cells, resulting in dysfunction.

### 4.3 Ovarian fibrosis

The ovaries of patients with PCOS are characterized by thickening owing to greater collagen deposition and fibrosis (64). Transforming growth factor (TGF)-β is the key factor that drives fibrosis in various tissues (65, 66), and it has been reported that the serum concentration and ovarian expression of TGF-β1 are higher in patients with PCOS (67, 68). Both TGF-β1 and connective tissue growth factor (CTGF) in GCs have essential roles in extracellular matrix remodeling in the ovary (69–71), and it has also been shown that HA induces ovarian fibrosis *via* the TGF-β signaling pathway in a rat model (72, 73).

ER stress and TGF-β1 signaling in GCs are activated, and interstitial fibrosis is marked, in the ovaries of both patients and mice with PCOS (4). Furthermore, ER stress stimulates the expression of TGF-β1 and CTGF in cultured human GCs, and treatment with TUDCA has been shown to reduce ovarian fibrosis in mice with PCOS, which is accompanied by reductions in ER stress and TGF-β1 expression. The concentrations of TGF-β1 and CTGF are also high in the ovaries of rats with PCOS, but the administration of adrenomedullin reduces their ER stress and the ovarian concentrations of profibrotic factors (63). These findings suggest that ER stress contributes to ovarian fibrosis *via* the TGF-β1 signaling pathway in the pathology of PCOS. Taken together with the findings that ER stress causes the activation of TGF-β signaling and fibrosis in various organs (74–76), it is conceivable that ER stress activated by HA in the ovaries of women with PCOS is implicated in the etiology of the interstitial fibrosis that characterizes the ovaries of women with PCOS.

### 4.4 Abnormal ovarian steroid hormone metabolism

In patients with PCOS, it has been shown that steroidogenic gene expression is altered in GCs (77), and that the concentrations of estrogen and progesterone are low in both serum and follicular fluid (78–81). In addition to HA, ER stress affects both estrogen and progesterone production by GCs (82, 83). The concentrations of both hormones are lower in the culture medium of GCs derived from patients with PCOS than in that from control patients, and these concentrations are increased by TUDCA treatment, in association with a reduction in ER stress (42). The expression of the ovarian steroidogenic genes Cyp19a1 and Cyp11a1 is high in the ovaries of rats with PCOS (43). In addition, the expression of aryl hydrocarbon receptor (AHR) and Cyp1b1, which is a downstream target of AHR and metabolizes estrogen, is high in the GCs of patients and mice with PCOS (84). AHR is a well-established receptor for endocrine-disrupting chemicals (EDCs), which accumulate in the ovaries of women with PCOS, but it also plays diverse roles in metabolic, developmental, and pathologic processes (85–87). In the ovary, it has been demonstrated that the AHR signaling pathway is involved in follicular development and estradiol biosynthesis (88). ER stress increases the expression of AHR and Cyp1b1 in cultured human GCs, and the administration of an AHR inhibitor ameliorates the abnormal reproductive phenotype in mice with PCOS, including their estrous cyclicity and atretic follicle counts (85). These findings suggest that ER stress in the follicular microenvironment contributes to the disruption of steroid hormone metabolism as part of the pathophysiology of PCOS.

### 4.5 Cumulus oocyte complex expansion

Ovulatory dysfunction is one of the key phenotypes of PCOS. Ovulation is a complex process, during which the maturation of the oocyte and its release from an ovarian follicle occur in synchrony with dynamic tissue remodeling, including the expansion of cumulus oocyte complex (COC) (89–91).

The Notch pathway, which regulates cell fate *via* cell-to-cell juxtacrine interaction, is involved in various physiological and pathological processes, such as organogenesis and carcinogenesis (92–95). Notch signaling in the follicular microenvironment also regulates ovarian development, including follicular assembly and growth, steroidogenesis, and angiogenesis (34, 96, 97). Notch signaling is activated in the GCs of patients and mice with PCOS (98). The UPR activates Notch signaling followed by the induction of some ovulation-related genes in cultured human GCs. ER stress increases the expansion of cultured murine COCs through Notch signaling. Moreover, cumulus oocyte complex (COC) expansion is upregulated in mice with PCOS, which is attenuated by administration of a Notch signaling inhibitor. It has also been reported that ER stress and androgens induce the expansion of cultured murine COCs, and that this is reduced by treatment with TUDCA or metformin (99). These findings indicate that ER stress and the resulting activation of Notch signaling interferes with ovulation in PCOS.

### 4.6 Accumulation of advanced glycation end-products in the ovary

Advanced glycation end-products (AGEs) are endogenously produced through the Maillard reaction between sugars and the free amino groups of proteins or other substrates (100), and are exogenously obtained in the diet and through smoking (101, 102). AGEs are proinflammatory factors and are involved in several diseases, including diabetes, metabolic syndrome, and cardiovascular disease. Patients with PCOS have high concentrations of Advanced glycation end-products (AGEs) in their serum and ovaries (103–105). AGEs bind to cell membrane receptors for AGEs (RAGE) and activate several proinflammatory signaling pathways, causing follicular growth arrest and ovulatory dysfunction (104, 105). ER stress induced by androgens is associated with increases in the expression of AGEs and RAGE in cultured human GCs (106). The administration of TUDCA to mice with PCOS reduces the accumulation of AGEs and the expression of RAGE and restores estrous cyclicity and follicular development. Thus, the HA associated with PCOS causes the accumulation of AGEs in the ovary by activating ER stress, resulting in follicular growth arrest.

## 5 Summary and future perspectives

**Figure 2:**
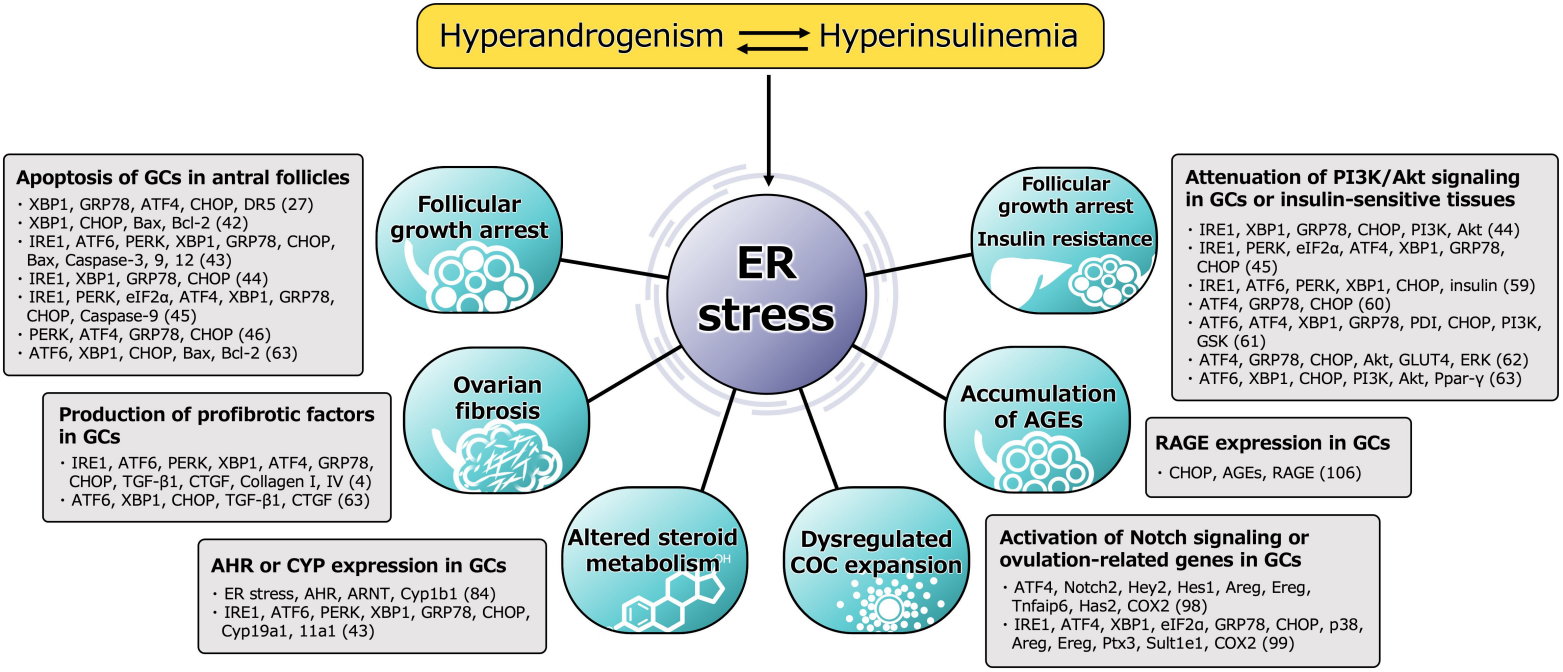
Functional alteration of GCs induced by ER stress in pathophysiology of PCOS. Molecules involved in the mechanism, either directly or indirectly activated by ER stress, are shown. The number in parentheses refers to the reference number of citation. ER stress is activated in the follicular microenvironment by HA and IR, two key factors underpinning the heterogenous etiology of PCOS. ER stress and the subsequent activation of the UPR contribute to the pathophysiology of PCOS by impairing the function of GCs in multiple ways. ER stress activates the apoptotic cascade in GCs and is associated with follicular growth arrest. The UPR branches, PERK-ATF4-CHOP or IRE1-TRAF2-JNK, induce several caspases-dependent apoptosis. ER stress also induces the ovarian fibrosis that is characteristic of PCOS by increasing the production of profibrotic cytokines, such as TGF-β1 and CTGF, in GCs *via* the UPR pathway, especially involved in XBP1. ER stress is also associated with high expression of AHR, AHR nuclear translocator (ARNT) and its downstream Cyp1b1 in GCs, which causes alterations in ovarian steroid hormone metabolism. In addition to this, other steroidogenic enzymes, such as Cyp19a1 and Cyp11a1, are induced by the UPR pathway. ER stress also perturbs COC expansion *via* activation of ATF4 and Notch signaling followed by the induction of ovulation-related genes, such as amphiregulin (Areg), epiregulin (Ereg), hyaluronan synthase 2 (Has2), tumor necrosis factor alpha-induced protein 6 (Tnfaip6) and cyclooxygenase 2 (COX2), in GCs, which might also contribute to the ovulatory dysfunction. ER stress is also associated with the accumulation of AGEs in GCs and an increase in expression of the RAGE through induction of CHOP, resulting in a failure of follicular development. In addition, ER stress in the pancreas, liver, muscle, and adipocytes induces IR and compensatory hyperinsulinemia, which aggravate HA and directly contribute to ovarian dysfunction by impairing PI3K/Akt signaling in GCs. Thus, a vicious circle is formed by HA, hyperinsulinemia, and other proinflammatory factors, and these factors are connected through ER stress.

ER stress is activated in the follicular microenvironment by HA and IR, two key components of the heterogenous etiology of PCOS. ER stress and the resulting activation of the UPR contribute to the pathophysiology of PCOS by disturbing the function of GCs in multiple ways (**Figure 2**). ER stress activates the apoptotic cascade in GCs and is associated with follicular growth arrest. It also induces ovarian fibrosis, which is a characteristic feature of PCOS, and is mediated through greater production of profibrotic cytokines, such as TGF-β1, by GCs. ER stress also induces the expression of AHR and activates downstream signaling in GCs, causing abnormal ovarian steroid hormone metabolism. It also perturbs COC expansion by activating Notch signaling in GCs, which might underpin the ovulatory dysfunction. The accumulation of AGEs in GCs is caused by ER stress through higher RAGE expression, which results in follicular growth arrest. In addition, ER stress in the pancreas, liver, muscle, and adipocytes induces IR and compensatory hyperinsulinemia, which worsen the HA and directly contribute to ovarian dysfunction. Thus, a vicious circle is formed by HA, hyperinsulinemia, and other proinflammatory factors, and these are connected by ER stress.

These findings indicate that ER stress represents a promising therapeutic target for PCOS. TUDCA and 4-PBA are chemical chaperones that directly reduce ER stress and have been clinically used for the treatment of liver disease and urea cycle disorders, respectively. Several natural compounds, including inositol, resveratrol, curcumin, and traditional Chinese medicines, have also been used to treat PCOS either clinically or experimentally, and it was shown that reductions in ER stress at least in part explain their effects, although the precise underlying mechanisms have not been determined (107–109). Lifestyle modification plays a role in treatment of PCOS (8), and the ER may represent a target of this, together with other local factors in the follicular microenvironment, including oxidative stress, inflammation, and the accumulation of AGEs. In addition, it would be interesting to evaluate the relationship between the follicular microenvironment and the gut microbiome, which is closely associated with lifestyle and plays a causative role in pathogenesis of PCOS (110, 111).

## 6 Conclusion

The activation of ER stress in the follicular microenvironment of patients with PCOS forms part of a vicious circle with other local factors, including high levels of androgens, oxidative stress, and inflammation, as well as with systemic features of PCOS, including IR. The abnormal follicular microenvironment causes multiple defects in GCs, contributes to the pathogenesis of PCOS, and closely interacts with the systemic features of PCOS. Further research regarding the targeting of ER stress would be useful for the development of treatments of this enigmatic syndrome that are based on its pathological mechanism.

## Author contributions

HK and MH contributed to conception and design of the work and drafting of the article. AK, ZX, TT, NS, CK, JA, NT, and YU contributed to critical revision of the intellectual content of the work. YO supervised the work and contributed to critical revision of the intellectual content of the work. All authors contributed to the article and approved the submitted version.
